# Dioxin and Dioxin-like Compounds and Human Health

**DOI:** 10.3390/toxics11060512

**Published:** 2023-06-06

**Authors:** Muneko Nishijo

**Affiliations:** Department of Public Health, Kanazawa Medial University, Uchinada 920-0293, Ishikawa, Japan; ni-koei@kanazawa-med.ac.jp

In epidemiological studies, associations of dioxin and dioxin-like (dl)-compound exposure with metabolic diseases, including diabetes and metabolic syndrome, in adults and with neurodevelopmental problems and earlier/later puberty in children have been suggested in the general population and in environmentally exposed populations.

2,3,7,8-Tetrachlorodibenzo-p-dioxin (TCDD) is the most toxic polychlorinated dibenzo-p-dioxins and -furan (PCDD/F) congener and is a by-product of the production of herbicides such as 2,4,5-trichloroacetophenoxy acetic acid (2,4,5-T). Workers in Germany, Netherland, Bohemia (former Czechoslovakia), and USA were accidentally exposed to TCDD in chemical plants producing 2,4,5-T including TCDD between the 1950s and 1970s. A large-sized population including workers and residents in Seveso, Italy was exposed to extremely high levels of TCDD caused by an industrial accident in 1976. In Missouri, USA, a significant amount of TCDD-contaminated waste oil was sprayed in residential areas in the period 1971–1972, resulting in environmental contamination of TCDD. Portland Harbor, a section of Willamette River flowing through the Willamette Valley in Oregon, USA, has been industrialized and impacted by urban and industrial activities for over a century. This area is heavily contaminated by a variety of chemicals including polychlorinated biphenyls (PCBs), PCDD/Fs, and polycyclic aromatic hydrocarbon (PAH), which exert their effect through the aryl hydrocarbon receptor (AHR).

All over southern Vietnam, the U.S. military sprayed millions of liters of herbicide such as Agent Orange, which is composed in part by 2,4,5-T, during Operation Ranch Hand (1961–1971), and not only Vietnamese residents and solders but also American and Korean soldiers were exposed to TCDD. Even after more than 40 years since the war ended, high dioxin levels, particularly high TCDD levels, have been found in the soil and sediment inside former U.S. air bases, particularly Da Nang, Phu Cat, and Bien Hoa airbases, which caused contamination of the environment and human health, including workers and residents living around the airbases.

This Special Issue includes a total of 10 articles, including 6 original articles and 3 reviews, aiming to provide recent or overall study results to investigate the effects of dioxins on human health, focusing on children and adults exposed to historical and/or present pollution in Vietnam. One more original article in the present Special Issue is a study showing that coupling environmental whole mixture toxicity screening with unbiased RNA-seq using biological responses in Danio rerio (zebrafish) is a useful method to evaluate the toxicity of a mixture of compounds with AHR, such as samples at numerous sites in the Portland Harbor Superfund Site.

Articles by Pham-The et al. [1] and Tran et al. [2] in this issue were results of follow-up studies of children from the Da Nang cohort in Vietnam. Da Nang airbase, located in central Vietnam, is one of a number of former U.S. airbases contaminated with dioxins as a result of the use of Agent Orange and other herbicides. In 2007–2008, we previously measured levels of 17 PCDD/F congeners in the breast milk of mothers residing nearby Da Nang airbase; significantly, we found that these levels were 3–4 times higher than those of mothers living in unsprayed areas, suggesting that dioxin exposure originating from herbicide is still high enough to increase health risks in the residents living in nearby areas of Da Nang airbase.

Follow-up studies of this Da Nang birth cohort identified adverse effects of dioxin exposure on infant and child neurodevelopment from 4 months to 8 years of age, including increased autistic traits (poor social and communication abilities) associated with high TCDD exposure and poor language and motor development associated with high TEQ-PCDD/F exposure in boys at 3 years of age. Boys showed poorer cognitive ability associated with high TCDD exposure and poor coordination movement skills associated with TEQ-PCDD/Fs exposure at 5 years of age.

Regarding ADHD likelihood, Pham-The et al. [1] reported that hyperactivity scores, a subscale of ADHD rating scale, were significantly higher in boys with high TCDD at 5 years of age, but no association between ADHD symptoms and dioxin exposure was found in girls. In contrast, at 8 years of age, girls showed high hyperactivity scores in the high-TCDD group, which was also significantly associated with unusual behavior scores according to the Autism Spectrum Rating Scale (ASRS), suggesting high perinatal TCDD exposure may increase ADHD likelihood and autistic traits, particularly in girls.

At 8 years of age, boys with high TCDD showed increased reading learning difficulties, although no increase in ADHD symptoms was found. Tran et al. [2] summarized all study results from 4 months to 8 years of age and concluded that perinatal TCDD exposure impacts social–emotional cognitive functions, leading to sex-specific neurodevelopmental disorders, i.e., learning difficulty in boys and ADHD in girls.

Manh et al. [3] collected 45 blood samples from 9-year-old children living in areas nearby Phu Cat airbase, one of the former U.S. airbases, and 35 blood samples of 9-year-old children in the nonexposed area to make 12 pooled samples, and they measured 17 PCDD/F congeners in sera. The mean of the TEQ of PCDD/Fs level in Phu Cat was more than three times higher than that in the nonexposed area, but no TCDD was detected even in Phu Cat samples. The serum levels of some congeners, but not TEQ-PCDD/Fs, were correlated with those in breast milk, which were collected from mothers when they were nursing their children.

Pham N.T. et al. [4] investigated the effect of perinatal dioxin exposure (indicated by dioxins in breast milk) on the gaze behavior of 142 children at 3 years of age from the 2012 Bien Hoa birth cohort; the children, residing in the areas most contaminated areas by TCDD, originating from Agent Orange around Bien Hoa airbase in Vietnam, were examined via an eye-tracker. The gaze fixation duration on facial areas when viewing 10 still images of children was calculated as the gaze behavior index. The face fixation duration (%) significantly decreased as TCDD concentrations increased in a dose–effect manner in girls, which suggested atypical gaze behavior for watching human faces. Furthermore, these girls with atypical gaze behavior showed lower social communication scores according to the ASRS and smaller head sizes, suggesting increased autistic traits in girls.

Between 2007 and 2015, Nishijo et al. [5] collected a total of 861 breast milk samples (597 samples from 3 herbicide-sprayed areas including Quang Tri, Da Nang, and Bien Hoa in southern Vietnam; 264 samples from 3 unsprayed areas in northern Vietnam) and determined PCDD/Fs in each sample from mothers 1 month after delivery. The levels of TEQ-PCDD/Fs and 17 PCDD/F congeners were significantly higher in the sprayed area samples than the unsprayed area samples. The authors found particular PCDD/F congener patterns for different areas. High TCDD concentrations were found in Bien Hoa, high levels of TCDD and 1,2,3,6,7,8-hexaCDD were found in Da Nang, and high 1,2,3,4,6,7,8-heptaCDD levels were found in Quan Tri. High 1,2,3,4,7,8-hexaCDF and 1,2,3,4,6,7,8-heptaCDF concentrations were also found in Da Nang and Quang Tri. The associations between the levels of TCDD, 1,2,3,4,7,8-HexaCDF, and 1,2,3,4,6,7,8-HeptaCDF were different for samples from primipara and multipara mothers, suggesting that breast feeding affected PCDF concentrations more than PCDD concentrations.

Vu et al. [6] investigated associations between dioxin exposure and brain structural irregularities in 32 Vietnamese men living near Bien Hoa airbase. Two exposure markers were used: 1) blood dioxin levels as a marker of exposure in adulthood, and 2) perinatal dioxin exposure during pregnancy, estimated by a maternal residency in the areas around Bien Hoa airbase. All subjects underwent brain magnetic resonance imaging (MRI) scans. Correlations between regional grey matter volumes and blood dioxin levels and between brain regional volumes of men with and without perinatal dioxin exposure were determined by voxel-based morphometry (VBM). Blood TCDD was associated with a low volume of the medial temporal pole and fusiform gyrus. Levels of TEQ-PCDDs were correlated with low medial temporal pole volume. However, 1,2,3,4,7,8-HxCDD was associated with high middle frontal gyrus and cerebellum volume. In men with perinatal dioxin exposure, the left inferior frontal gyrus pars orbitalis volume was significantly lower than in those without perinatal exposure. These results suggest that dioxin exposure during the perinatal period and in adulthood may cause altered regional brain volume, which can lead to cognitive deficits and unusual social–emotional behavior.

Pham P.Q. et al. [7] collected liver biopsy samples for histopathological examination from 33 chronic hepatitis patients living around the Da Nang Airbase. They found that increased TCDD levels in blood were associated with increased levels of liver function markers such as aspartate aminotransferase (AST), alanine aminotransferase (ALT), protein and total bilirubin, and high liver fibrosis stages classified using the METAVIR fibrosis staging system for histopathological examination. Similarly, increased TEQ-PCDD/Fs levels were associated with higher levels of AST and protein and the liver fibrosis stage. These findings suggest TCDD exposure may influence liver cells to increase fibrosis leading to an increased risk of liver cancer, suggesting that regular health check-ups, particularly liver function tests and imaging examinations, should be required for all subjects living in dioxin contamination areas in Vietnam.

Takiguchi et al. [8] reviewed previous publications in which the effects of PCDD/Fs and dioxin-like PCBs on the teeth and bones of animals and humans were found to identify future research directions, particularly for epidemiological studies of populations exposed to PCDD/Fs in the environment. Previously, it has been reported that exposure of fetuses to PCDD/Fs may affect odontogenesis, particularly enamel formation in human and animals. However, the effects of PCDD/Fs on bone genesis are limited to palatine bone. Exposure to PCDD/Fs during infancy may affect both teeth and bones, but the effects on bones may be reversible. High PCDD/Fs exposure even during adulthood may adversely affect teeth in human and animals. In contrast, however, PCDD/Fs exposure may induce osteogenesis and improve bone properties because the disrupting effects of PCDD/Fs cause bone remodeling and vitamin D activation in animals. More studies involving humans are required to investigate previously found associations between the PCDD/F concentrations and biological markers for teeth and bones, including metabolites of vitamin D.

The aim of the review by Vuong [9] is to discover whether there is a relationship between dioxin exposure and cancer incidence in the hotspot regions of Vietnam by estimating the risk ratio index. The results of the study show that the incidence of cancer (soft tissue sarcoma; Hodgkin’s and non-Hodgkin’s lymphoma; lung, prostate, and liver cancer) in the dioxin-exposed Vietnamese population is much higher than that found in the results of studies published in other countries, as a result of the high levels of dioxins in southern Vietnam, where Agent Orange was sprayed during the Vietnam War. Further studies on the health effects of dioxins in the Vietnamese population, including cancer incidence, should be conducted with improved research methods.

Rude et al. [10] analyzed the toxicity of passive sampling device extracts from two points (river mile 6.5 W and 7 W) in the Portland Harbor Superfund Site in Oregon, USA, which has been industrialized and heavily contaminated by a variety of chemicals including PCBs, PCDD/Fs, and PAH, using coupling environmental whole mixture toxicity screening with unbiased RNA-Seq. The toxicity was evaluated using developmental toxicity assays in Danio rerio (zebrafish) shown as “wavy” notochord malformation. The gene expression from two extracts was parallel, although it was more evident in river mile 6.5 W than 7.0 W. Differential expression, reminiscent of the wavy notched phenotype, was not accounted for by either class of chemicals. Therefore, these techniques offer a compelling method for non-targeted hazard characterization of whole mixtures without requiring complete chemical characterization.
